# Supplementary material for: The Effect of AlI3 Nanoadditive on the Thermal Behavior of PMMA Subjected to Thermoanalytical Py-GC-MS Technique
Source: Materials (Basel). 2021 Nov 19;14(22):7036. doi: 10.3390/ma14227036 (PMC8624407; doi:10.3390/ma14227036)
Supplement: Supplementary file 1 [file materials-14-07036-s001.zip › materials-1439867-supplementary.pdf]

materials

Supplementary Material

The Effect of AlI3 Nano-additive on the Thermal Behavior of PMMA Subjected to Thermoanalytical Py-GC-MS Technique

Muhammad Adnan [1], Taj Ur Rahman [1,*], Ali Bahadur [2,*], Muhammad Aurang Zeb [1,*], Wajiha Liaqat [1], Takashiro Akitsu [3], Shams H. Abdel-Hafez [4] and Wael A. El-Sayed [5]

[1] Department of Chemistry, Mohi-Ud-Din Islamic University, Nerian Sharif, Azad Jammu & Kashmir.
[2] Department of Transdisciplinary Studies, Graduate School of Convergence Science and Technology, Seoul Na-tional University, Seoul, 08826, South Korea.
[3] Department of Chemistry, Faculty of Science Division II, Tokyo University of Science, Tokyo, Japan
[4] Department of Chemistry, College of Science, Taif University. P.O.Box 11099, Taif 21944. Saudi Arabia
[5] Department of Chemistry, College of Science, Qassim University, Buraida, Saudi Arabia
* Correspondence: alibahadur138@snu.ac.kr (A. B), taj_urrehman81@yahoo.co.uk (T. U. R), muham-mad_au-rangzeb@hotmail.com (M.A.Z).

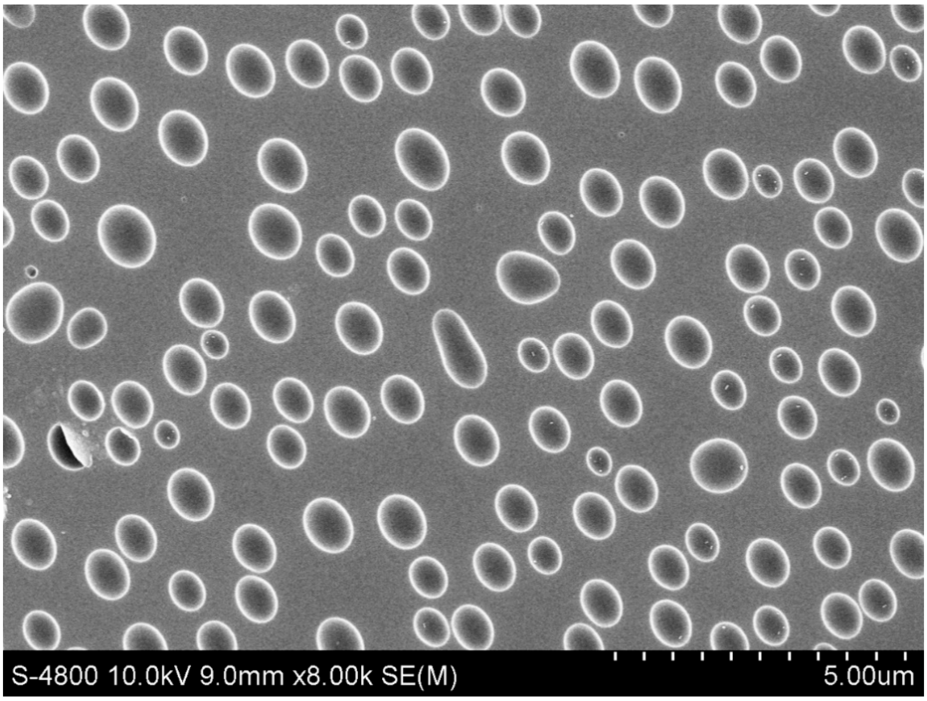

Figure S1. SEM images of M5 composite.

Table S1. Kinetic data of composite M1-M5.

Sample	Activation E (kJ/mol)	Order
M	19.71	1
M1	9.52	2
M2	8.26	2
M3	5.82	2
M4	6.34	2
M5	5.41	2
AlI_3	4.36	1

Table S2. The horizontal burning rate for PMMA composite.

Composite	Burning time (s)	Length of strip (mm)	Burning rate (mm/s)
M	15	100	6.66
M1	51	100	1.96
M2	53	100	1.88
M3	56	100	1.78
M4	61	100	1.63
M5	67	100	1.49
